# Supplementary material for: Small noncoding RNA interactome capture reveals pervasive, carbon source–dependent tRNA engagement of yeast glycolytic enzymes
Source: RNA. 2023 Mar;29(3):330–45. doi: 10.1261/rna.079408.122 (PMC9945440; doi:10.1261/rna.079408.122)
Supplement: Supplemental Material [file supp_29_3_330__DC1.html]

Small non-coding RNA Interactome Capture reveals pervasive, carbon source-dependent tRNA engagement of yeast glycolytic enzymes — Small noncoding RNA interactome capture reveals pervasive, carbon source–dependent tRNA engagement of yeast glycolytic enzymes — Supplemental Material 

# Small noncoding RNA interactome capture reveals pervasive, carbon source–dependent tRNA engagement of yeast glycolytic enzymes

## Supplemental Material

- Supplemental\_Figure\_S1.ai
- Supplemental\_Figure\_S2.ai
- Supplemental\_Figure\_S3.ai
- Supplemental\_Figure\_S4.ai
- Supplemental\_Figure\_S5.ai
- Supplemental\_Table1\_RIC2C\_Limma\_results\_V1.xlsx
- Supplemental\_Table2\_CLIP2C\_gene.xlsx
- Supplemental\_Table3\_snRIC2C\_Limma\_results.xlsx
