# Supplementary figures and images for: Small noncoding RNA interactome capture reveals pervasive, carbon source–dependent tRNA engagement of yeast glycolytic enzymes
Source: RNA. 2023 Mar;29(3):330–45. doi: 10.1261/rna.079408.122 (PMC9945440; doi:10.1261/rna.079408.122)

Figure S1

**A**

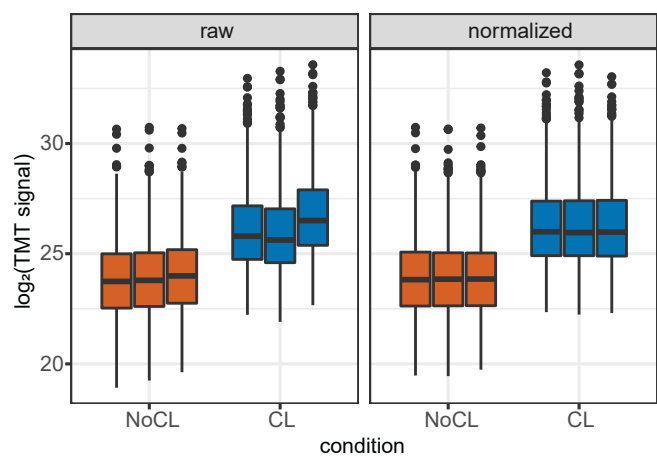

**B**

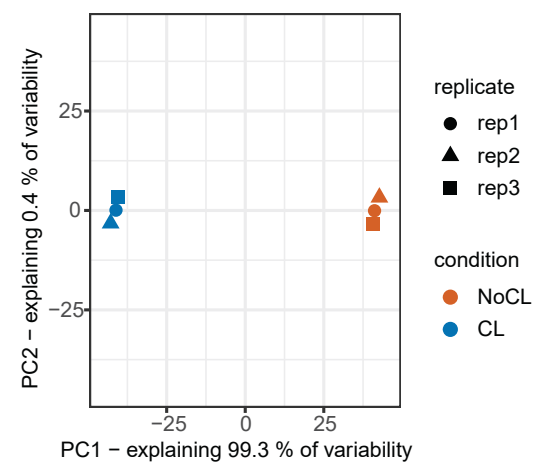

Supplement: Supplemental Material [file supp_079408.122_Supplemental_Figure_S1.ps]

Figure S2

**A**

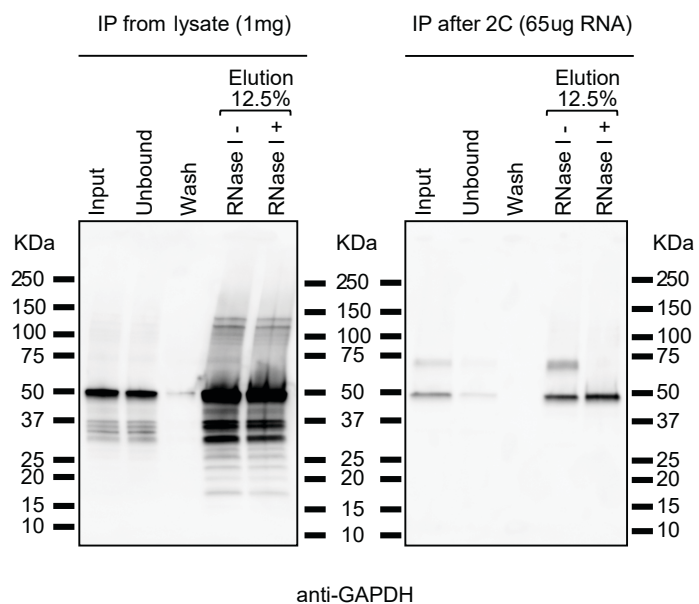

**B** High exposure of A

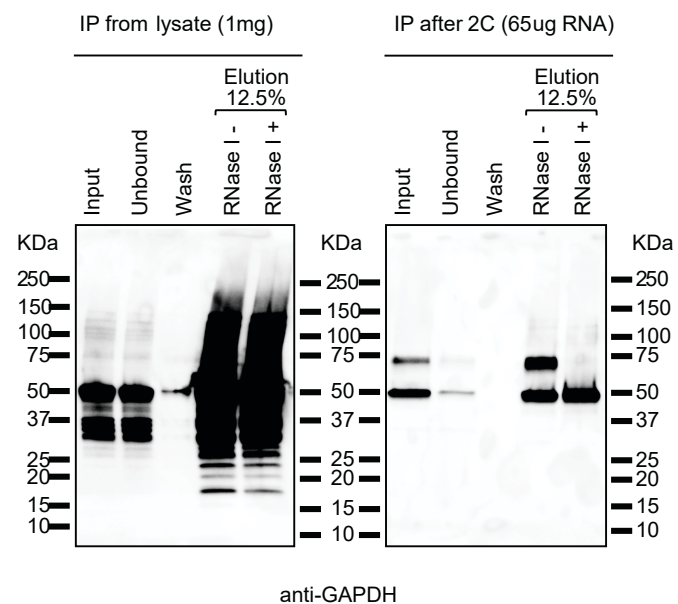

Supplement: Supplemental Material [file supp_079408.122_Supplemental_Figure_S2.ps]

Figure S3

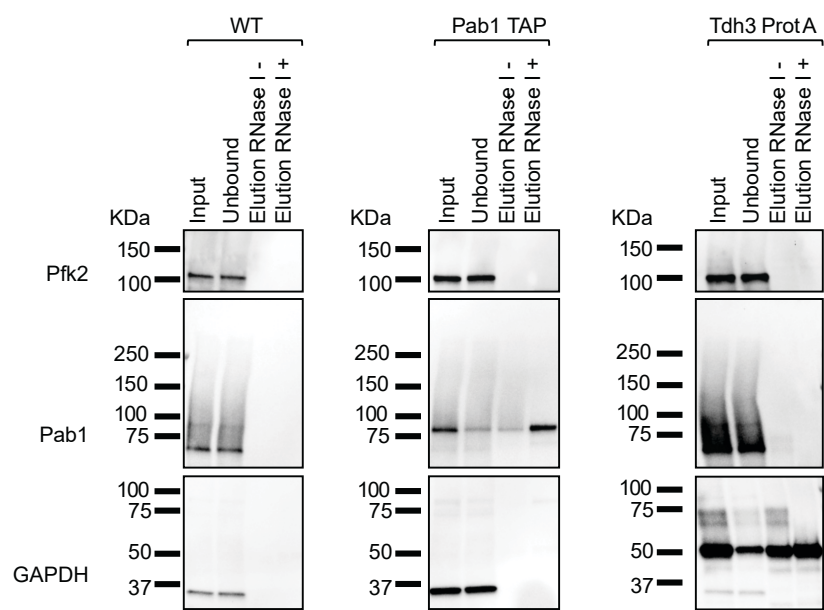

Supplement: Supplemental Material [file supp_079408.122_Supplemental_Figure_S3.ps]

Figure S4

A

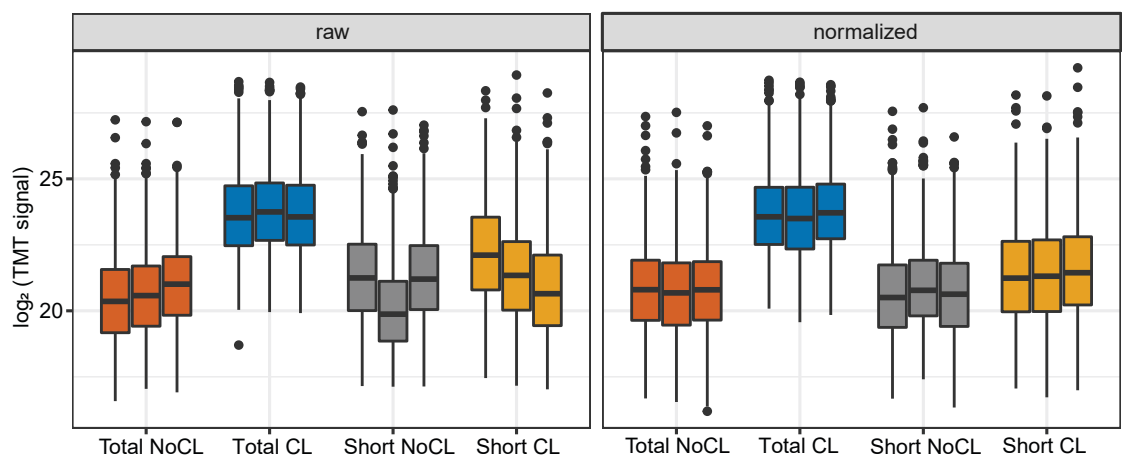

B

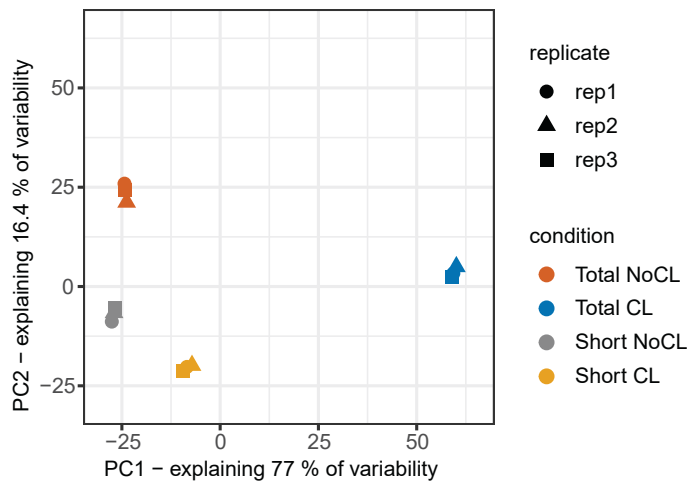

C

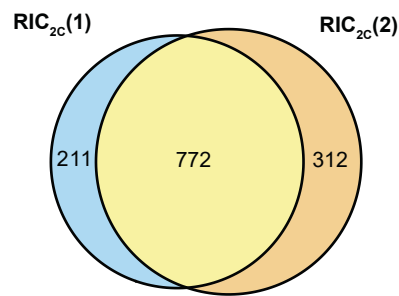

Supplement: Supplemental Material [file supp_079408.122_Supplemental_Figure_S4.ps]

Figure S5

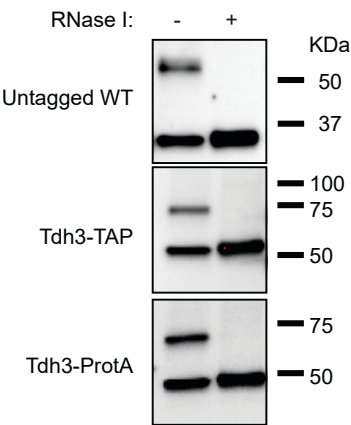

Supplement: Supplemental Material [file supp_079408.122_Supplemental_Figure_S5.ps]
